# Hepatic Farnesoid X-Receptor Isoforms α2 and α4 Differentially Modulate Bile Salt and Lipoprotein Metabolism in Mice

**DOI:** 10.1371/journal.pone.0115028

**Published:** 2014-12-15

**Authors:** Marije Boesjes, Vincent W. Bloks, Jurre Hageman, Trijnie Bos, Theo H. van Dijk, Rick Havinga, Henk Wolters, Johan W. Jonker, Folkert Kuipers, Albert K. Groen

**Affiliations:** 1 Department of Pediatrics, University Medical Center Groningen, University of Groningen, Groningen, The Netherlands; 2 Department of Laboratory Medicine, University Medical Center Groningen, University of Groningen, Groningen, The Netherlands; IRCCS Istituto Oncologico Giovanni Paolo II, Italy

## Abstract

The nuclear receptor FXR acts as an intracellular bile salt sensor that regulates synthesis and transport of bile salts within their enterohepatic circulation. In addition, FXR is involved in control of a variety of crucial metabolic pathways. Four FXR splice variants are known, *i.e.* FXRα1-4. Although these isoforms show differences in spatial and temporal expression patterns as well as in transcriptional activity, the physiological relevance hereof has remained elusive. We have evaluated specific roles of hepatic FXRα2 and FXRα4 by stably expressing these isoforms using liver-specific self-complementary adeno-associated viral vectors in total body FXR knock-out mice. The hepatic gene expression profile of the FXR knock-out mice was largely normalized by both isoforms. Yet, differential effects were also apparent; FXRα2 was more effective in reducing elevated HDL levels and transrepressed hepatic expression of Cyp8b1, the regulator of cholate synthesis. The latter coincided with a switch in hydrophobicity of the bile salt pool. Furthermore, FXRα2-transduction caused an increased neutral sterol excretion compared to FXRα4 without affecting intestinal cholesterol absorption. Our data show, for the first time, that hepatic FXRα2 and FXRα4 differentially modulate bile salt and lipoprotein metabolism in mice.

## Introduction

Bile salts are synthesized from cholesterol by well-characterized biosynthetic pathways (see [1] for review). The neutral pathway starts with 7α-hydroxylation of cholesterol by cholesterol 7α-hydroxylase (Cyp7a1) and the acidic pathway is initiated by sterol 27-hydroxylase (Cyp27a1). Newly synthesized primary bile salts are cholate (CA) and chenodeoxycholate (CDCA) in humans, whereas in rodents CDCA is rapidly converted into the more hydrophilic muricholates (MCA). Sterol 12α-hydroxylase (Cyp8b1) is required for the biosynthesis of CA and determines the ratio CA to CDCA. The chemical diversity of bile salts is further expanded by conjugation to either taurine or glycine and by the actions of intestinal bacteria *via* deconjugation, oxidation of hydroxyl groups and dehydroxylation, creating a bile salt pool with specific physiochemical properties. In addition to their well-established functions in generation of bile formation by the liver and absorption of dietary fats and fat-soluble vitamins from the intestine, bile salts are now recognized to act as ‘integrators of metabolism’ [1], [2].

Bile salts contribute to control of a broad range of metabolic pathways, including their own synthesis in liver and transport within the enterohepatic circulation, *via* activation of the Farnesoid X Receptor (FXRα/NR1H4, referred to as FXR) [3], [4]. FXR, a member of the nuclear hormone receptor superfamily, has four splice variants in humans and rodents [5], [6]. These isoforms arise from a single gene through alternative splicing of exon 5 and the use of distinct promoters that initiate transcription from either exon 1 or exon 3. The different promoters of the *FXR* gene initiate expression of either FXRα1 and FXRα2 or of FXRα3 and FXRα4 transcripts. FXR isoforms show differences in spatial and temporal expression as well as in transcriptional activity [6]. FXRα1 and FXRα3 transcripts contain four additional amino acids in the hinge domain, adjacent to the DNA-binding domain, which make these two isoforms less transcriptionally active compared to FXRα2 and FXRα4. In mice all four isoforms are abundantly expressed in liver, while FXRα3 and α4 are abundantly expressed in ileum, moderately in kidney and at low levels in stomach, duodenum, and jejunum. Furthermore, FXRα1 and α2 are moderately expressed in ileum and adrenal gland [6].

In the liver, bile salt-activated FXR suppresses, amongst others, Cyp7a1 expression, in a Short Heterodimer Partner(SHP/NR0B2)- and Liver Receptor Homolog-1 (LRH-1/NR5A2)-dependent manner [7], [8]. Also Cyp8b1 and Cyp27a1 are regulated by FXR, in a SHP- and Hepatocyte Nuclear Factor-4α (HNF4A)-dependent manner [9], [10]. In the past decade, FXR has emerged as an important regulator of lipid as well as glucose homeostasis and to have anti-inflammatory properties [1]. Hence, FXR is considered a promising target for treatment of a number of metabolic and liver diseases. Treatment with synthetic FXR agonists showed improved insulin sensitivity in diabetic mouse models [11], [12] and prevented formation of atherosclerotic plaques in atherogenic-prone mice [13]. Furthermore, administration of the semi-synthetic obeticholic acid (OCA) increased insulin sensitivity and reduced markers of liver inflammation and fibrosis in patients with type 2 diabetes and nonalcoholic fatty liver disease [14].

In light of the increasing evidence for the therapeutic potential of FXR agonists in the regulation of lipid metabolism, we addressed the specific roles of FXRα2 and FXRα4, the transcriptionally most active isoforms of FXR [5], [6], on bile salt and lipid metabolism *in vivo* by means of a liver-specific FXRα2 or FXRα4 expressing mouse model. Our data revealed some distinct functions of FXRα2 and FXRα4 in control of plasma cholesterol levels; only hepatic FXRα2 expression could rescue the elevated plasma HDL levels of FXR KO mice. Moreover, FXRα2 and FXRα4 were found to differentially regulate Cyp8b1 expression *in vivo*, which coincided with compositional changes in the respective bile salt pools. Our data show, for the first time, that hepatic FXRα2 and FXRα4 differentially modulate bile salt and lipoprotein metabolism in mice.

## Materials and Methods

### Construction and production of self-complementary AAV vectors

The coding sequences of murine FXRα1, FXRα3, HNF4α, SHP and RXRα were amplified using specific primer pairs harboring a Kozak consensus ATG initiation codon and a *BamH1* restriction side at the 5′ end and a *Not1* restriction side at the 3′ end (S1 Table) [15]. As a template source, cDNA was synthesized from hepatic RNA from C57BL/6J wild-type mice. The products were cloned into pcDNA5/FRT/TO (Invitrogen, Life Technologies, Bleiswijk, The Netherlands). The presence of the correct gene was sequence-verified. Subsequently, the MYTG insertion was deleted from FXRα1 and α3 using inverse PCR primers, to generate FXRα2 and FXRα4. Self-complementary AAV (scAAV) serotype 8 vectors with the liver specific LP1 promoter driving murine FXRα2 or murine FXRα4 expression were generated by replacing the factor IX of scAAV2-LP1-hFIXco [16], [17] using *Pme1*, *EcoR1* and *Hpa1*. Additionally, AAV8- pseudotyped vectors were made using the packaging plasmid pAAV8-2. Production, purification and titration of all vectors were performed as described [18], [19].

### Cell culture, transfections and reporter assays

CV1 cells (a kind gift from Ronald M. Evans, Salk Institute, San Diego, USA) were maintained in DMEM (Gibco, Breda, The Netherlands) supplemented with 10% FCS (Sigma Aldrich Chemie BV, Zwijndrecht, The Netherlands), 100 U/ml penicillin and 100 µg/ml streptomycin (Gibco, Breda, The Netherlands). Cultures were maintained at 37°C and 5% CO2 in a humidified incubator. Cells were transiently transfected using FuGENE 6 transfection reagent (Promega, Leiden, The Netherlands) according to manufacturer’s protocol. Cell lysis and luciferase assays were performed using a dual luciferase reporter assay system (Promega, Leiden, The Netherlands) according to manufacturer’s protocol. Briefly, the human PGL4-SHP (CHR1_M0312_R1, Switchgear Genomics, Menlo Park, CA, USA) and Cyp8b1 [20] (a kind gift from John Y. Chiang, Northeastern Ohio Medical University, Rootstown, USA) promoter reporters were cotransfected together with the pcDNA5/FRT/TO murine FXR isoforms and the heterodimer RXR alpha/NR2B1 for 48 hrs. Cells were treated with 50 µM CDCA 24 prior to cell lysis when stated.

### Animal experiments

FXR knock-out mice [21] backcrossed on a C57BL/6J background and C57BL/6J wild-type mice were housed individually in a temperature- and light-controlled facility with 12 hours light-dark cycling and received food and water *ad libitum*. Experiments were approved by the Ethical Committee for Animal Experiments of the University of Groningen. Age-matched male FXR knock-out received 1×10^11^ AAV vector genomes or PBS injected into the retro-orbital sinus. PBS-injection, rather than AAV-injected was used as control since GFP from our AAV-GFP induced several responses, which was already know from previous studies [22], [23]. When indicated, mice received chow (RMH-B, Hope Farms, Woerden, The Netherlands) supplemented with 0.5% (w/w) cholate (Calbiochem, La Jolla, CA, USA) in the final week before termination.

### Experimental procedures

Four weeks after the scAAV injections, cholesterol fluxes were measured as described previously [24]. Briefly, at day 0 mice received an intravenous dose of 0.3 mg (0.73 µmol) cholesterol-D7 (Cambridge Isotope Laboratories, Inc, Andover, MA, USA) dissolved in Intralipid (20%, Fresenius Kabi, Den Bosch, The Netherlands) and an oral dose of 0.6 mg (1.535 µmol) cholesterol-D5 (Medical Isotopes, Inc, Pelham, NH, USA) dissolved in MCT oil (Pharmacy UMCG, Groningen, The Netherlands). Blood spots were collected from the tail daily for 10 days. At the end of the experiment, mice were anesthetized and after puncturing the gallbladder and disposal of its contents, hepatic bile was collected for 20 minutes from the common bile duct *via* the gallbladder [25]. Tissues were excised and feces was collected from individual mice for 72 hours prior to termination.

### Analytical procedures

Hepatic lipids were extracted according to Bligh & Dyer [26]. Plasma and liver triglycerides, total cholesterol and free cholesterol contents were determined using commercially available kits (Roche Diagnostics, Mannheim, Germany and DiaSys Diagnostic Systems, Holzheim, Germany). Plasma levels of alanine aminotransferase (ALAT) and aspartate aminotransferase (ASAT) were determined using commercially available kits (Spinreact, Santa Coloma, Spain). Biliary phospholipid content was determined according to Böttcher *et al.* [27]. Cholesterol in bile was measured according to Gamble *et al.* [28]. Pooled plasma samples were subjected to fast protein liquid chromatography (FPLC) gel filtration using a Superose 6 column (GE Healthcare, Little Chalfont, UK). Biliary and fecal bile salt composition were quantified using capillary gas chromatography (Hewlett-Packerd gas chromatograph; HP 6890) equipped with a FID and a CP Sil 19 capillary column; length 25 m, internal diameter 250 µm and a film thickness of 0.2 µm (Chrompack BV, Middelburg, The Netherlands). Bile salts were methylated with a mixture of methanol and acetyl chloride and trimethylsilylated with piridyne, N, O-Bis(trimethylsilyl) trifluoroacetamide and trimethylchlorosilane. The murine bile salt species include cholate (CA), deoxycholate (DCA), chenodeoxycholate (CDCA), α-muricholate (α-MCA), β-muricholate (β-MCA), ω-muricholate (ω-MCA), hyodeoxycholate (HDCA), lithocholate (LCA) and ursodeoxycholate (UDCA). We consider cholate and deoxycholate as CA-derived bile salts and the others as CDCA-derived bile salts. Fecal cholesterol and its derivatives (also known as neutral sterols) were trimethylsilylated with pyridine, N, O-Bis (trimethylsilyl) trifluoroacetamide and trimethylchlorosilane and measured on the GC equipped with the same column. Cholesterol enrichment was determined by capillary gas chromatography on a Agilent gas chromatograph (7890A; Amstelveen, The Netherlands) equipped with a 30 m×0.25 mm column, with a film thickness of 0.25 µm (ZB-5; Bester, Amstelveen, The Netherlands) connected to a Agilent mass spectrometer (5975C). Cholesterol was derivatized with N, O-Bis (trimethylsilyl) trifluoroacetamide containing 5–10% trimethylchlorosilane. Isotope ratios were determined in the selected ion monitoring mode on m/z 458 (M0) to 465 (M7).

### RNA isolation and measurement of mRNA levels by quantitative real-time PCR

Tissue samples for isolation of RNA were snap frozen in liquid nitrogen and stored at minus 80°C. Samples were homogenized and total RNA using TRI-Reagent (Sigma, St. Louis, MO, USA). RNA concentration was determined using the Nanodrop spectrophotometer (NanoDrop 2000c, Thermo Scientific Inc., Walham, MA, USA). cDNA was obtained from total RNA using the RT procedure using Moloney-Murine Leukemia Virus (M-MLV) reverse transcriptase (RT) (Invitrogen, Life Technologies, Bleiswijk, The Netherlands) with random primers. Gene expression was measured with a 7900HT FAST system using FAST PCR mix, Taqman probes and MicroAmp FAST optical density 96-well plates (Applied Biosystems Europe, Nieuwekerk ad IJssel, The Netherlands). PCR results of liver and intestine were normalized to *36b4* mRNA levels. Primer and probe sequences can be found at (http://www.RTprimerDB.org).

### Explorative Illumina microarray analysis

For explorative microarray analysis, total hepatic RNA was prepared from separate groups of 0.5% cholate-fed FXRα2- or FXRα4-transduced FXR knock-out mice (n = 6 per group) using TRI-reagent (Sigma-Aldrich, St. Louis, MO, USA). RNA quality and concentration was assessed with an Biorad Experion Bioanalyzer. Starting with 200 ng of RNA, with an RNA Quality Indicator of at least 8. RNA was amplified and labeled using the Illumina TotalPrep RNA Amplification Kit (Applied Biosystems, Nieuwekerk ad IJssel, The Netherlands). The WG-6 v2 expression arrays, containing 45281 transcripts (Illumina, San Diego, USA), were processed according to the manufactures protocol and slides were scanned immediately. Quality control, normalization (quantile), batch correction (Combat), prefiltering (fold change 1.1) and statistics (IBMT) were performed in MADMAX [29]. A list of significant changed annotated genes between FXRα2 (n = 6) and FXRα4 (n = 6), including False Discovery Rates (FDR)-corrected p-values (10%) was generated. All microarray data reported are described in accordance with MIAME guidelines and are available in the GEO database (http://www.ncbi.nlm.nih.gov/geo/query/acc.cgi?acc=GSE51805). Identification of overrepresented functional categories among responsive genes and their grouping into functionally related clusters was performed using the DAVID Functional Annotation Clustering tool [30].

### Western blotting for FXR protein expression

Livers were homogenized (15% w/w) in buffer containing 50 mM Tris (pH 7.4), 300 mM sucrose, 10 mM EDTA, 10 mM DTT and Complete (Roche Diagnostics, Almere, The Netherlands). Protein was determined using BCA protein assay (Pierce Biotechnology, Rockford, IL, USA). Total liver homogenates (25 µg protein) for detection of FXR were electrophoresed through 7% polyacrylamide gels and blotted on Hybond ECL membranes (Amersham, Little Chalfont, UK). Membranes were blocked in phosphate-buffered saline (pH 7.4) containing 0.1% Tween 20 and 4% skim milk powder. Membranes were incubated with anti-human FXR mouse antibody (clone no. A9033A, Perseus Proteomics Inc., Tokyo, Japan) or anti-actin (Sigma-Aldrich, St. Louis, MO, USA). After washing, immunocomplexes were detected using horseradish peroxidase-conjugated goat anti-mouse IgG2a (Southern Biotech, Uithoorn, The Netherlands) or Horseradish peroxidase conjugated goat anti-rabbit IgG (Santa Cruz Biotechnology, Delaware, CA, USA) and SuperSignal West Dura substrate (ThermoScientific, Rockford, IL, USA).

### Statistics

Data are shown as Tukey’s Box-and-Whiskers plots or average ± standard deviations. Statistical analysis was assessed using Kruskal-Wallis H test followed by Conover post-hoc comparison or students t-test using Brightstat [31].

## Results

### Characterization of the mouse model

**Figure 1:**
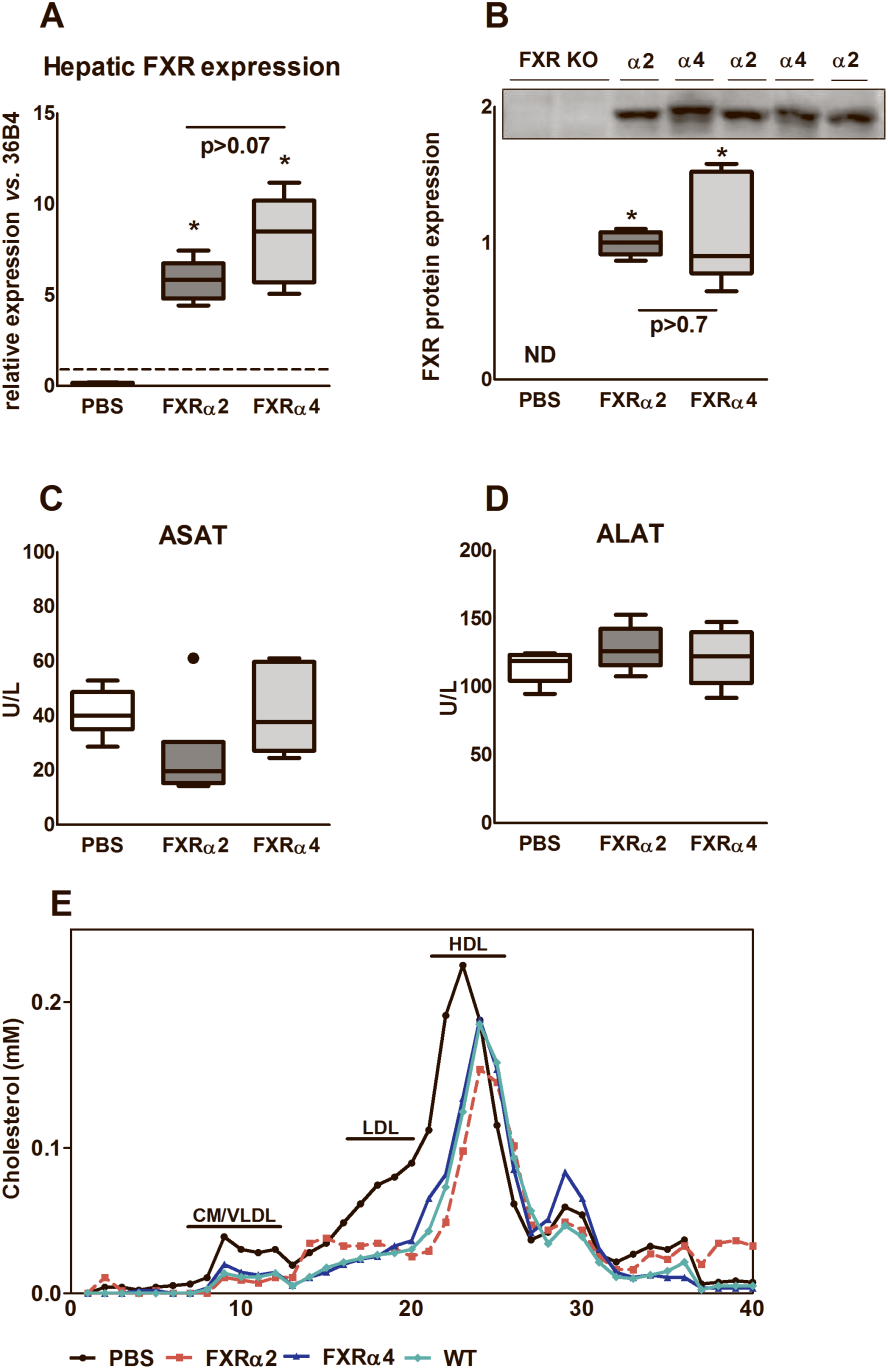
Characterization of the PBS-injected and the liver-specific scAAV-FXRα2 or scAAV-FXRα4 transduced FXR knock-out mice. Hepatic FXR gene expression (A) and protein (B) levels in total PBS-injected FXR knock-out mice *versus* stably transduced scAAV-FXRα2 or scAAV-FXRα4 FXR knock-out mice. Dotted line in (A) represents WT FXR expression levels. Plasma ASAT (C) and ALAT (D) levels were not altered in the AAV-treated mice compared to PBS-injected controls. (E) Cholesterol distribution in FPLC-separated lipoprotein fractions. Values are presented in box-and-whisker plots or averages (n = 4–6 animals per group). *p<0.05 *vs.* PBS.

At first we established the diurnal rhythmicity and spatial expression of the four murine FXR isoforms. Bile salt synthesis shows a strong circadian rhythm [1], [32], but mRNA levels of hepatic FXR isoforms did not show any diurnal variation (S1A Figure). Most prominent differences in expression between the isoforms were observed in skeletal muscle, intestine and kidney (S1B Figure), confirming previous reports [5], [6], while hepatic expression of FXR isoforms was comparable. To directly compare the physiological roles of hepatic FXRα2 and FXRα4, we generated self-complementary adeno-associated virus particles (scAAV) expressing the coding regions of murine FXRα2 or FXRα4 behind the liver-specific fatty acid-binding protein 1 (LP1) promoter. Hepatic FXR mRNA expression (Fig. 1A) and protein levels (Fig. 1B) were similarly induced by scAAV particle injection. Compared to PBS-injected controls, plasma ASAT and ALAT levels were unchanged in particle-injected mice, implying that stable scAAV transduction did not negatively impact on liver cell integrity (Fig. 1C/D).

### FXR isoforms differentially modulate lipid metabolism

The potency of hepatic FXRα2 and FXRα4 to normalize the characteristic plasma lipid profile of FXR knock-out mice [21], [33] was evaluated first. Both isoforms reduced the elevated total plasma cholesterol levels (table 1) seen in PBS-injected FXR KO, FXRα2 being more effective than FXRα4 in this respect, but neither FXRα2 nor FXRα4 completely normalized cholesterol levels to those seen in wild-type mice. FPLC-profiling revealed reduced cholesterol levels in the VLDL-, LDL- and particularly HDL-sized fractions of FXRα2- and FXRα4-expressing mice compared to PBS-injected controls and wild-type mice (Fig. 1E), with FXRα2 showing the most pronounced reduction of HDL-cholesterol. Both FXRα2 and FXRα4 caused a lowering of plasma triglyceride (TG) levels, yet, wild-type levels were not reached in either case.

**Table 1:** Animal characteristics under chow-fed conditions.

	FXR^+/+^		FXR^−/−^	
Morphometric parameters	WT	PBS	FXRα2	FXR4
Body weight (g)	28.0 (26.0–28.9)	27.2 (25.0–30.5)	27.1 (24.0–28.7)	26.5 (25.4–27.6)
Liver weight (% BW)	4.9 (4.5–5.5)	5.5 (4.9–6.0)	5.1 (4.3–5.5)	5.0 (4.8–5.3)
**Plasma**				
triglycerides (mmol/l)	0.6 (0.5–1.0)	1.4 (1.0–2.6)*	1.1 (0.9–1.2)* #	0.8 (0.7–1.6)* #
cholesterol (mmol/l)	2.6 (1.9–3.1)	7.6 (7.2–7.9)*	3.7 (3.4–3.9)* #	5.8 (5.4–6.5)* ^#$^
**Liver**				
triglycerides (µmol/g)	10.3 (6.6–12.6)	13.6 (7.6–15.9)	7.4 (6.5–9.0)* #	8.7 (7.6–12.2)
cholesterol (µmol/g)	7.1 (6.5–8.1)	5.1 (4.7–5.4)*	6.1 (5.7–6.9)* #	6.3 (5.9–7.9)* #
**Biliary output**				
bile flow (ml/day/100 g)	14.8 (12.9–19.5)	14.6 (10.7–16.6)*	19.2 (17.2–24.0)* #	14.6 (12.4–17.8)* ^#$^
bile salts (µmol/day/100 g)	435.1 (262.4–518.3)	482.6 (291.8–778.4)	309.3 (258.9–612.9)	405.7 (338.1–475.4)
cholesterol (µmol/day/100 g)	8.5 (6.4–13.2)	8.1 (5.7–8.8)	10.8 (8.4–11.7)	9.1 (7.0–11.8)
phospholipids (µmol/day/100 g)	76.6 (69.3–91.6)	79.9 (72.9–91.1)	100.6 (82.6–122.6)* #	98.3 (84.3–148.3)* #

Animal characteristics of chow-fed FXR knock-out mice injected with PBS or stably transduced with hepatic-specific scAAV-FXR2 or scAAV-FXR4 were compared to chow-fed wild-type (WT) mice. Values are presented as median (range) (n = 5–6 animals per group).

*p<0.05 *vs.* WT,

# p<0.05 *vs* PBS,

$ p<0.05 between isoforms.

### FXR isoforms differentially modulate bile salt composition

**Figure 2:**
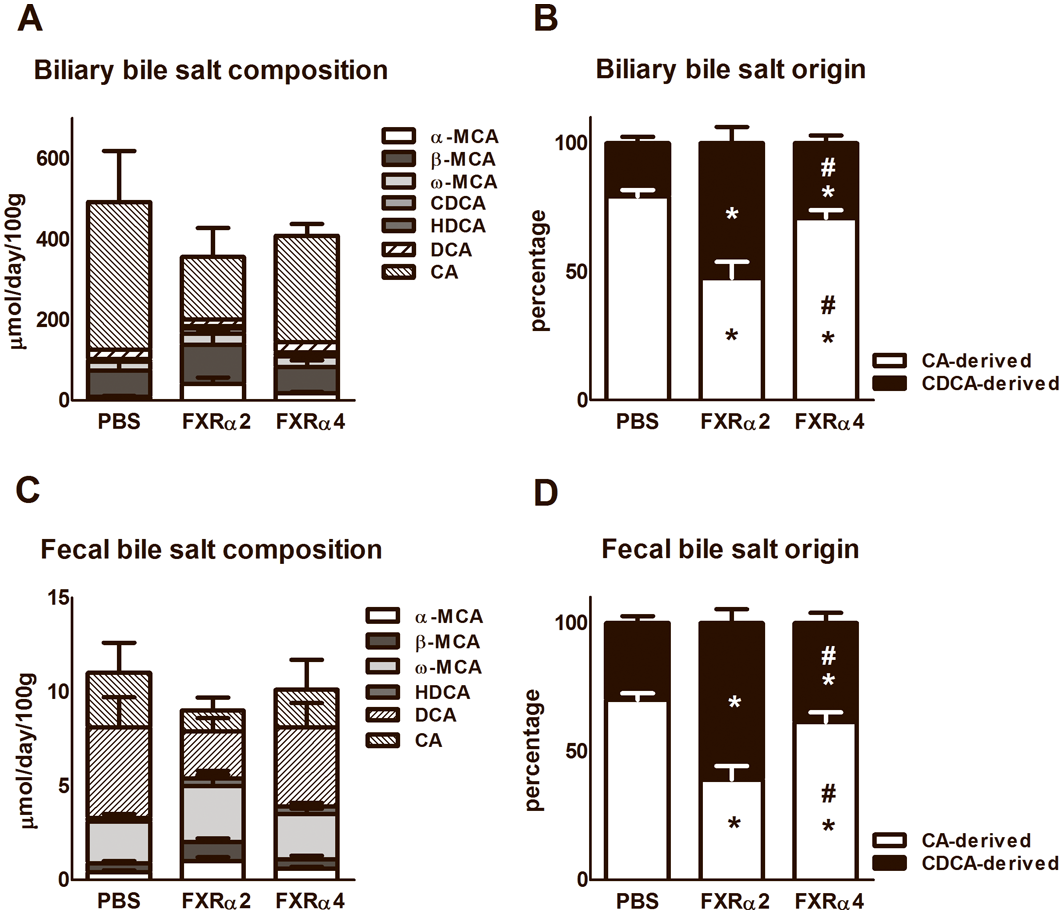
Physiochemical properties of the bile salt pool. Bile was canulated for 20 minutes and feces was collected 72 hr prior to termination of chow-fed PBS-injected and the liver-specific scAAV-FXRα2 or scAAV-FXRα4 transduced FXR knock-out mice. Total biliary (A) and fecal (C) bile salt composition are similar between 3 groups. The biliary (B) and fecal (D) bile salt origins show differences between the 3 groups. (α-MCA; α-muricholate, β-MCA; β-muricholate, ω-MCA; ω-muricholate, CDCA; chenodeoxycholate, HDCA; hyodeoxycholate, DCA; deoxycholate, CA; cholate. We consider cholate and deoxycholate as CA-derived bile salts and the others as CDCA-derived bile salts. Values are presented as average ± standard deviations (n = 6 animals per group). *p<0.05 *vs.* PBS; ^#^P<0.05 between FXR isoforms.

Next, we tested whether the physiochemical properties of the bile salt pool were differentially affected by FXRα2 and FXRα4. The total amount of biliary and fecal bile salt excretion was, surprisingly, not different between groups (Fig. 2A and C). Yet, the bile salt profile in FXRα2-expressing mice showed a shift towards CDCA-derived muricholates relative to CA-derived bile salts that were more prominent in FXRα4-expressing mice (Fig. 2B and D). Specifically, biliary CA and fecal DCA were reduced in the first, while the relative and absolute abundances of α-MCA, CDCA and HDCA were increased (S2 Table). These data imply a shift in bile salt synthesis towards the production of relatively hydrophilic muricholates in FXRα2-expressing mice. Based on similar biliary and fecal bile salt excretion rates, the latter reflecting hepatic bile salt synthesis under steady sate, it can be assumed that the sizes of circulating bile salt pools remained unaffected upon expression of either FXRα2 or FXRα4.

### Non-biliary cholesterol excretion is enhanced in FXRα2-transduced mice

**Figure 3:**
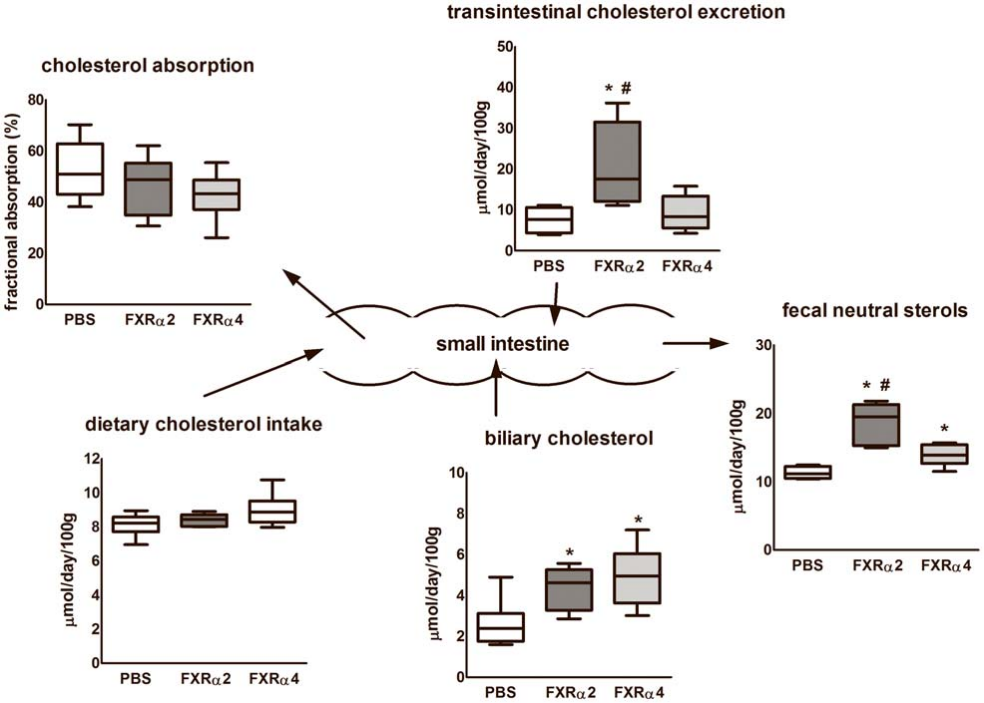
Cholesterol balance. PBS-injected and the liver-specific scAAV-FXRα2 or scAAV-FXRα4 transduced FXR knock-out mice were subjected to cholesterol kinetics using isotope labeled tracers to calculate the contribution of dietary, biliary and intestinal cholesterol to fecal sterols. Values are presented in box-and-whisker plots (n = 6 animals per group). *p<0.05 *vs.* PBS; ^#^P<0.05 between FXR isoforms.

Since muricholates are more hydrophilic than CA is, we tested whether the differences in the hydrophobicity of the bile salt pool between FXRα2- and FXRα4-expressing mice affect intestinal cholesterol absorption. Fecal neutral sterol output was increased in FXRα2 mice compared to PBS controls and FXRα4 mice (Fig. 3). This increase was not due to diminished fractional cholesterol absorption or increased biliary cholesterol secretion, since these were similar between groups. Hence, the differences in fecal sterols must come directly from the intestine, *i*.*e*., reflect the transintestinal cholesterol excretion pathway (TICE) [34], [35]. Hepatic FXRα2 expression increased this pathway by 100% on average. Thus, FXRα2-expressing mice showed induction in fecal neutral sterol output and TICE without altering intestinal cholesterol absorption efficiency.

### FXR isoforms differently regulate primary bile salt biosynthesis genes

To gain insight in the modes of action by which hepatic FXRα2 and FXRα4 act on metabolic processes, an explorative microarray was performed on the livers of 0.5% cholate-fed FXR KO mice transduced with scAAV-FXRα2 or scAAV-FXRα4. Cholate-feeding was employed to obtain supraphysiological activation of the FXR isoforms. A total of 720 probes representing 556 coding genes were identified to represent differentially expressed genes in liver (microarray data can be obtained from: http://www.ncbi.nlm.nih.gov/geo/query/acc.cgi?acc=GSE51805; listed top-15 genes; S3 Table). Broad scale KEGG pathway analysis [30] revealed, as expected, that the primary bile salt biosynthetic pathway pops up within the top 5 of most relevant pathways. Compared to expression levels seen in the livers of FXRα4-transduced mice, FXRα2-transduced mice showed lower expression levels of *Cyp7a1* (19% reduction) and *Cyp8b1* (17% reduction), indicating that FXRα2 transrepressed both *Cyp7a1* and *Cyp8b1* more strongly than FXRα4. Since *Cyp8b1* activity is the major determinant of CA biosynthesis in the neutral branch of bile salt synthesis, the data support the notion that hepatic FXRα2 particularly inhibits the CA production. On the other hand, FXRα4-transduced mice showed lower expression of *Cyp7b1* (60% reduction). Since *Cyp7b1* is involved in CDCA biosynthesis via the so called acidic or alternative branch of bile salt synthesis, these data indeed indicate that hepatic FXRα4 particularly inhibits CDCA/MCA production, as expected on the basis of analysis of biliary and fecal bile salt composition (S2 Table). Also *Cyp17a1*, a C21 steroidogenic enzyme, was differentially regulated by the FXR isoforms (62% reduction in FXRα2 *versus* FXRα4). Some of the differentially expressed genes as well as FXR itself and its targets *Shp* and *Abcb11/Bsep* were confirmed using quantitative PCR together with some intestinal FXR targets, in which the differences were even more pronounced (table 2). Consistent with the shift towards CDCA-derived muricholates relative to CA-derived bile salts in FXRα2-expressing mice, hepatic *Cyp8b1* mRNA expression was reduced compared to FXRα4-expressing mice (reduction of 33%) and PBS-injected FXR KO (reduction of 60%). CYP7B1, the enzyme of the alternative bile salt biosynthetic pathway, was only down-regulated in the liver of FXRα4-expressing mice on 0.5% cholic acid-feeding. The expression of *Abcb11/Bsep* also showed differential regulation by the two FXR isoforms on mRNA level. FXRα2-expressing mice showed higher *Abcb11/Bsep* mRNA expression compared to FXRα4 in both conditions. Bile flow in these animals was increased compared to FXRα4-expressing and control mice (table 1), yet, secretion of endogenous bile salts was unaffected implicating stimulation of bile salt-independent bile flow. Surprisingly, the intestinal FXR target genes *Fgf15* and *Fabp6*/*Ibabp* were affected in the FXRα2- and FXR4α-transduced mice on both diets (table 2), indicating a cross-talk between liver and intestine.

**Table 2:** Hepatic and intestinal expression profile of genes involved in bile salt metabolism.

	chow-fed			0.5% cholic acid-fed		
*Liver*	PBS	FXRα2	FXRα4	PBS	FXRα2	FXRα4
Nr1h4/Fxr**	1.0±0.2	36.1±7.1*	50.0±14.9*	1.0±0.5	31.9±7.7*	29.8±10.8*
Nr0b2/Shp	1.0±0.3	1.8±0.8	1.4±1.0	1.0±0.7	5.0±0.8*	4.1±1.0*
Abcb11/Bsep	1.0±0.2	2.9±0.2*^#^	2.1±0.5*	1.0±0.3	6.7±0.9*^#^	3.8±0.8*
Cyp7a1	1.0±0.4	0.7±0.3	0.6±0.3	1.0±0.7	0.02±0.02	0.2±0.1
Cyp7b1	1.0±0.4	0.9±0.2	1.1±0.2	1.0±0.4	0.9±0.2	0.3±0.0*^#^
Cyp8b1	1.0±0.2	0.4±0.1*^#^	0.6±0.3*	1.0±0.4	0.01±0.01*^#^	0.03±0.01*
***Intestine***	**PBS**	**FXRα2**	**FXRα4**	**PBS**	**FXRα2**	**FXRα4**
Nr1h4/Fxr**	1.0±0.2	0.7±0.1	0.8±0.1	1.0±1.0	23.5±7.7	20.9±17.5
Fgf15	1.0±0.7	0.4±0.3*	0.4±0.3*	1.0±0.9	2.9±1.1*	1.5±1.0
Fabp6/Ibabp	1.0±0.3	3.0±0.9*	2.3±0.9*	1.0±0.9	2.4±1.0*	1.2±0.5*

Hepatic and intestinal gene expression determined by quantitative real-time PCR of genes involved in synthesis, transport and signaling of bile salts in chow- and 0.5% cholic acid-fed PBS-injected and stably transduced scAAV-FXRα2 or scAAV-FXRα4 FXR knock-out mice. Gene expression levels were normalized to 36b4 and corrected to expression levels of PBS-injected FXR knock-outs. Values are presented as average ± standard deviations (n = 5–6 animals per group). *p<0.05 *vs*. PBS; ^#^p<0.05 between FXR isoforms. ** note that our QPCR primers pairs always picked up FXR signal, even in FXR knock-outs. Expression levels were normalized to expression levels found in PBS-injected FXR knock-out mice.

### FXR isoforms differentially induce SHP *in vitro*

**Figure 4:**
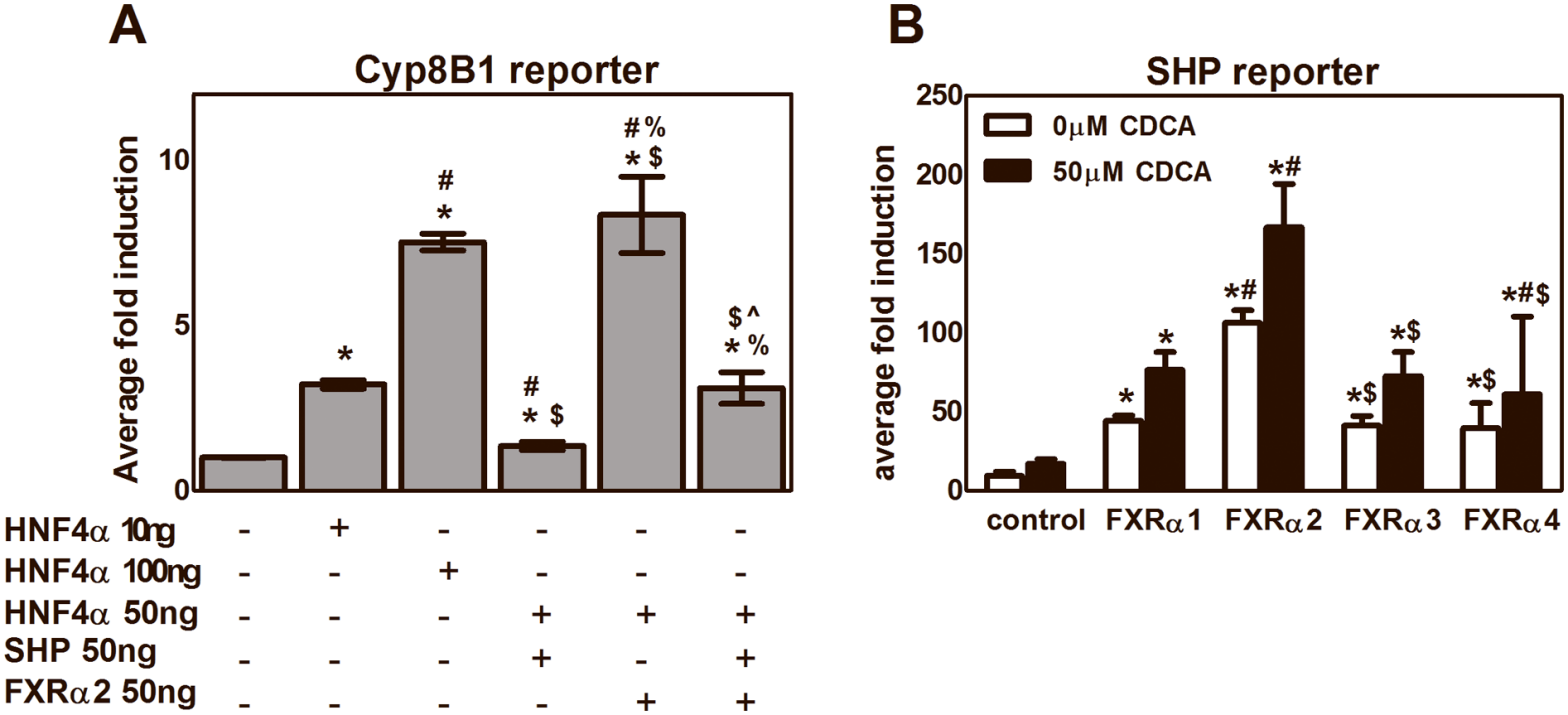
FXR-mediated transreppression of Cyp8b1 and transcriptional activity of the FXR isoforms *in vitro.* Luciferase assays where performed in CV1 cells using an luciferase reporter containing tandem copies of the FXRE from the Cyp8b1 (A) and SHP (B) genes. Activity is shown in fold induction, with the transfection efficiencies being normalized using a dual Renilla-Firefly luciferase assay. Activity is compared to empty vector control. Using the SHP receptor, cells were treated 24 hr post transfection either with 50 µM CDCA or vehicle. Data are presented as average ± standard deviations (n≥2). Significance Cyp8B1 reporter: *p<0.05 *vs*. control; #p<0.05 *vs.* HNF4α 10 ng; $p<0.05 *vs*. HNF4α 100 ng; &p<0.05 *vs* HNF4α and SHP; ∧p<0.05 *vs*. HNF4α and FXRα2. Significance SHP reporter: *p<0.05 *vs*. control; #p<0.05 *vs*. FXRα1; $p<0.05 *vs*. FXRα2; &p<0.05 *vs,* FXRα3.

In attempt to explain the differential effects of FXRα2 and FXRα4 on *Cyp8b1* expression, we have screened the promoter region of the *Cyp8b1* gene for FXREs using Mathinspector [36]. This screening revealed an IR1 FXRE in the promoter region of murine *Cyp8b1* starting at 99 base pairs from the transcription start site. Also in the human CYP8B1 promoter region an IR1 FXRE was found, at >2500 base pairs from the transcription start site. Unlike expected, we were unable to show direct FXR-mediated regulation of *Cyp8b1* expression in promoter studies, since FXRα2 could not transrepress the HNF-4α-activated Cyp8b1 reporter (Fig. 4A). However, we did find that FXR isoforms differentially induce *Shp* (Fig. 4B). *Shp* is a well-established FXR target gene whose product represses nuclear hormone receptor-mediated transactivation. Hence, this finding might indicate FXR-Shp-mediated Cyp8b1 transrepression, possibly explaining the changes in bile salt composition observed in the *in vivo* situation. FXR isoforms showed differences in their transcriptional activity (FXRα2>α4>α1>α3) independent of ligand activation, which was already shown in previous studies for *Abcb11/Bsep* but not for *Shp* [5], [6]. Compared to other isoforms FXRα2 showed an up to 50% increase in transcriptional activity upon activation using CDCA as ligand. However, the differences in hepatic *Shp* expression *in vivo* between chow-fed FXRα2- and FXRα4-expressing mice failed to reach statistical significance and therefore the physiological relevance, if any, of isoform-specific Shp regulation awaits further studies.

## Discussion

The most important finding of this study is it the demonstration, for the first time, of physiologically relevant differences in *in vivo* activity between the two transcriptionally most active FXR isoforms, *i.e*., FXRα2 and FXRα4. Our data demonstrate that FXRα2 and FXRα4 differentially affect transcriptional control of bile salt and lipoprotein metabolism in mice.

One of the phenotypic hallmarks of FXR knock-out mice concerns the plasma lipid profile with high levels of HDL-cholesterol and concomitantly elevated VLDL-cholesterol and triglyceride levels [11]. In our model, a first biological read-out was the (partial) normalization of this profile. Both FXR isoforms reduced HDL- and VLDL-cholesterol levels. However, total plasma cholesterol levels in FXRα2-expressing mice were markedly reduced compared to FXR KO values, while FXRα4 was less effective. In both situations, cholesterol levels found in wild-type mice were not reached, yet, the results underscore the role of hepatic FXR in control of plasma HDL levels. The existence of relationships between bile salt and lipid metabolism has been recognized for many years. The production of triglyceride-rich VLDL-particles, as well as their clearance from the circulation have been proposed to be influenced by FXR activity through modulation of hepatic *apoc2/apoc3* expression [37], [38] as well as VLDLR expression in cells [39]. On the other hand, bile salt-activated FXR lowers HDL-cholesterol, which has been attributed to suppression of *Apoa1* expression [40] and, very recently, to suppression of *CETP* expression which is, however, not expressed in wild-type mice [41]. The explorative microarray data of 0.5% cholic acid-fed FXRα2- and FXRα4-expressing mice revealed small but significant differences in hepatic *Apoc2* (20% upregulated in FXRα2) and *Vldlr* (30% upregulated in FXRα4) expression (S3 Table). Although relatively small, these differences indicate differential regulation of VLDL production as well as clearance from the circulation. Recently, the triglyceride-lowering capacity of FXR was linked to synthesis of 12α-hydroxylated bile salts *via* Foxo1, a key regulator of glucose metabolism [42]. Murine Foxo1 ablation caused elevated liver and plasma triglyceride levels, two signature lipid abnormalities of diabetes and the metabolic syndrome. These changes were associated with a relative deficiency of 12α-hydroxylated bile salts and in expression of *Cyp8b1*. In diabetes, the impaired insulin response led to dysfunctional Foxo1, which drives bile salt synthesis towards 12α-hydroxylated bile salts, leading to an abnormally hydrophilic bile salt pool affecting cholesterol and triglyceride metabolism. These data coincide with those observed in our model: FXRα2-expressing mice showed a stronger transrepression of *Cyp8b1* compared to FXRα4, thereby generating less 12α-hydroxylated bile salts and their plasma triglyceride levels tended to be higher than in FXRα4-expressing mice (table 1). Furthermore, FPLC data revealed slightly lower HDL-cholesterol in FXRα2-expressing mice compared to those expressing FXRα4. Thus, FXRα2 was found to target primarily the neutral branch of the bile salt biosynthesis pathway by transrepressing *Cyp7a1* and *Cyp8b1*, thereby inhibiting CA production. FXRα4, on the other hand, was found to particularly suppress the acidic branch of the bile salt biosynthesis pathway *Cyp7b1*, targeting primarily CDCA and MCA synthesis.

The changes in expression levels were consistent with compositional changes in the bile salt species in bile as well as in feces. The changes in bile salt composition alter the physiochemical properties of the bile salt pool. FXRα2 mice generate a more hydrophilic bile salt pool compared to FXRα4. In the cholate-fed situation, *i.e*., a state of FXR activation, also *Cyp17a1* was found to be differentially expressed by FXRα2 and FXRα4. FXRα2-expressing mice transrepressed *Cyp17a1* 62%more compared to FXRα4. Recently, increased production of 17-hydroxy steroid metabolites, the product of *Cyp17a1*, have been associated with juvenile onset cholestasis [43]. We did not observe large differences in bile flow or the grade of liver damage between the FXR isoforms that might indicate onset of cholestasis.

In the bile salt pool of Cyp7a1- as well as Cyp8b1-knock-out mice, in which CA was largely replaced by MCA, fractional cholesterol absorption from the intestine was decreased [44] while the intestinal cholesterol synthesis was induced [45] as a rescue mechanism. Since we observed similar alterations in bile salt pool composition in the FXRα2 mice, we analyzed cholesterol kinetics [24] to determine whole body cholesterol balance. Our data show increased fecal neutral sterol output in FXRα2-expressing mice, without concomitant reduction of fractional cholesterol absorption. Since there was no difference in biliary cholesterol secretion between FXRα2 and α4 -expressing mice, increased TICE accounted for the difference in neutral sterol excretion. The question arises how FXRα2 induces this effect. Our data show a differential effect of the FXR isoforms on plasma lipoprotein and bile salt pool distribution. Regulatory effects of plasma lipoproteins on the rate of TICE have not been described and the (lipoprotein) donor of TICE has still not been identified [46], [47]. The influence on the hydrophobicity of the bile salt pool may be more important. In vitro studies have shown that the presence of bile salts in the intestinal lumen, particular when combined with phospholipids, strongly stimulate this pathway but a clear influence of bile salt hydrophobicity was not demonstrated. A clear association between bile salt hydrophobicity and cholesterol absorption has been observed [48]. However, apparently in the present study the changes in the hydrophobicity of the bile salt pool were too subtle to affect cholesterol absorption. Hence it is not inhibition of cholesterol absorption that induces the increase in TICE but another as yet unidentified factor must be involved. We do speculate, however, that the change in the hydrophobicity of the bile salt pool drives the effect on TICE.

FXR regulates bile salt hydrophobicity *via* modulation of *Cyp7a1* and *Cyp8b1* expression through a SHP/LRH1- and HNF-4α-dependent pathway. To explain our results we speculated that FXR would directly transrepress *Cyp8b1* without interference of SHP. Promoter region analysis using Mathinspector [36] revealed a FXRE (agggcaggaacct) in the promoter region of both murine and human CYP8B1. Reporter studies, however, did not confirm direct FXR-mediated regulation of *Cyp8b1* expression using our murine FXR isoforms. Although hepatic gene expression of SHP did not significantly differ between the FXR isoform-transduced mice *in vivo*, our *in vitro* studies did show differential induction of SHP by both FXR isoforms. SHP is an orphan nuclear receptor that is regulated by many other nuclear receptors and transcription factors involved in metabolism and cancer [49] and also has been shown to be involved in control of *Cyp8b1* expression [50]. SHP can repress transcription factor-mediated transactivation by inhibiting DNA binding, competing for binding of coactivators or recruitment of corepressors. Hepatic SHP mRNA expression shows circadian rhythmicity, regulated by the clock gene *Nr1d1*(*Rev-erbα)* [51]. Because of the interaction with many other transcription factors and the circadian rhythmicity, the *in vivo* regulation of SHP is complex. Our laboratory very recently demonstrated that human FXR regulates *SHP* expression through direct binding to an LRH-1 binding site, independent of the known IR-1 and LRH-1 sites [52], which underscores the fact that the exact mechanisms by which SHP is regulated *in vivo* remains enigmatic. The differences in transcriptional activation of SHP, *i.e*., FXRα2 being more effective than FXRα4, may contribute to the differences observed in hepatic *Cyp8b1* expression, resulting in the concomitant differences in the bile salt pools between FXRα2- and FXRα4-expressing mice. Yet, more studies are required to substantiate this suggestion.

The intriguing question remains why FXR isoforms exist and what their specific physiological roles in different tissues actually are. FXRα2 and FXRα4 differ within their N-terminal parts, *i.e*., a 37 amino acid extension of FXRα4 compared to FXRα2. This difference can cause conformational changes in the FXR protein and, thereby, influence the transcriptional activity and/or transactivation/repression capacity on target genes. A dozen transcriptional cofactors have been described to influence FXR transactivation [53], but actions specific for the specific FXR isoforms have not been described. The relative abundance of hepatic FXRα3,4 was increased in children suffering from progressive familial cholestasis type I [54]. Also in cholangiocarcinomas [55] and colorectal adenocarcinoma [56] the relative abundance of hepatic and intestinal FXRα3,4 was increased. Apparently, disease states can be associated with a switch in FXR isoform expression in the liver and intestine which may influence outcome of disease progression. Recently, Vaquero *et al.* [57] reported that activation of human FXR depends on the pattern of FXR isoform expression and bile salt composition. They showed that cell-specific pattern of FXR isoforms determine the overall tissue sensitivity to FXR agonists and may be involved in the differential response of FXR target genes to FXR activation. The present study shows that the FXR isoforms influence physiology differentially and although murine and human bile salt and lipoprotein metabolism differ from each other the differential influence of the isoforms on the bile salt synthesis pathways will probably be similar.

In conclusion, we show for the first time physiological evidence for differential roles of FXRα2 and FXRα4 in control of bile salt and lipoprotein metabolism in mice. This might, in light of the increasing evidence for therapeutic potential of FXR agonists, be of importance for design of treatment strategies for metabolic diseases.

## Supporting Information

S1 Figure **Spatial and tissue-specific expression profiles of FXR isoforms in mice.** Relative expression was measured in livers from mice sacrificed at different time point during the day (A), in different tissues at 13∶00 h (B). Gene expression levels were normalized to 36B4. Data are presented as average ± standard deviation (n = 6–7 animals per group). *p<0.05 between FXRα1/2 and FXRα3/4. Wat; white adipose tissue, bat; brown adipose tissue, liv; liver, skm; skeletal muscle, kid; kidney, int; small intestine.
(DOCX)

S1 Table **Primer list.** Overview of the conventional primers used for cloning the different murine nuclear receptors. The FXRα1, FXRα3, RXRα, SHP and HNF4α primers were used to clone the FXRα1, FXRα3, RXRα, SHP and HNF4α genes. The FXRα2/4 primers were designed as nested primers to delete the four amino insertion in the hinge region of FXRα1 and FXRα3 to generate FXRα2 and FXRα4, respectively.
(DOCX)

S2 Table **Biliary and fecal bile salt profiles.** The specific bile salts in bile and feces were determined by gas chromatography and are presented as µmol/day/100 g body weight as average ± standard deviation (n = 5–6). *p<0.05 vs. PBS-injected FXR KO; #p<0.05 between FXR isoforms.
(DOCX)

S3 Table **Differentially expressed genes of explorative Illumina microarray of livers of 0.5% cholic acid-fed stably transduced scAAV- FXRα2- and scAAV-FXRα4-FXR knock-out mice.** Livers of 0.5% cholic acid-fed stably transduced scAAV- FXRα2- and scAAV-FXRα4-FXR knock-out mice were used for explorative microarray analysis. Listed top-15 genes and interesting targets, which showed statistically significant differences with a False Discovery Rate (FDR) <10%. “Fold change” represents the fold difference in expression between livers of stably transduced scAAV-FXRα2-and scAAV-FXRα4 FXR knock-out mice (both n = 6). The genes represented in bold are involved in bile salts and lipid metabolism.
(DOCX)
