# Supplementary material for: Hepatic Farnesoid X-Receptor Isoforms α2 and α4 Differentially Modulate Bile Salt and Lipoprotein Metabolism in Mice
Source: PLoS One. 2014 Dec 15;9(12):e115028. doi: 10.1371/journal.pone.0115028 (PMC4266635; doi:10.1371/journal.pone.0115028)
Supplement: S1 Figure — Spatial and tissue-specific expression profiles of FXR isoforms in mice. Relative expression was measured in livers from mice sacrificed at different time point during the day (A), in different tissues at 13∶00 h (B). Gene expression levels were normalized to 36B4. Data are presented as average ± standard deviation (n = 6–7 animals per group). *p<0.05 between FXRα1/2 and FXRα3/4. Wat; white adipose tissue, bat; brown adipose tissue, liv; liver, skm; skeletal muscle, kid; kidney, int; small intestine. (DOCX) [file pone.0115028.s001.docx]

**Figure S1. Spatial and tissue-specific expression profiles of FXR isoforms in mice.**

Relative expression was measured in livers from mice sacrificed at different time point during the day (A), in different tissues at 13:00h (B). Gene expression levels were normalized to 36B4. Data are presented as average ± standard deviation (n=6-7 animals per group). *p < 0.05 between FXRα1/2 and FXRα3/4. Wat; white adipose tissue, bat; brown adipose tissue, liv; liver, skm; skeletal muscle, kid; kidney, int; small intestine.
