# Supplementary material for: Hepatic Farnesoid X-Receptor Isoforms α2 and α4 Differentially Modulate Bile Salt and Lipoprotein Metabolism in Mice
Source: PLoS One. 2014 Dec 15;9(12):e115028. doi: 10.1371/journal.pone.0115028 (PMC4266635; doi:10.1371/journal.pone.0115028)
Supplement: S1 Table — Primer list. Overview of the conventional primers used for cloning the different murine nuclear receptors. The FXRα1, FXRα3, RXRα, SHP and HNF4α primers were used to clone the FXRα1, FXRα3, RXRα, SHP and HNF4α genes. The FXRα2/4 primers were designed as nested primers to delete the four amino insertion in the hinge region of FXRα1 and FXRα3 to generate FXRα2 and FXRα4, respectively. (DOCX) [file pone.0115028.s002.docx]

**Table S2. Biliary and fecal bile salt profiles**

The specific bile salts in bile and feces were determined by gas chromatography and are presented as µmol/day/100g body weight as average ± standard deviation (n=5-6). * p<0.05 *vs.* PBS-injected FXR KO; ^#^ p<0.05 between FXR isoforms.

|  | **PBS** | **FXRα2** | **FXRα4** |
| --- | --- | --- | --- |
| **total biliary bile salts** | **492.4 ± 164.6** | **355.4 ± 133.6** | **408.3 ± 45.7** |
| α-muricholic acid | 8.2 ± 3.2 | 40.4 ± 16.4* | 17.8 ± 3.6*^#^ |
| deoxycholic acid | 23.8 ± 7.7 | 15.8 ± 9.8 | 24.8 ± 10.6 |
| cholic acid | 365.9 ± 126.6 | 155.1 ± 72.1* | 264.1 ± 29.1^#^ |
| chenodeoxycholic acid | 4.0 ± 1.2 | 12.4 ± 4.7* | 6.0 ± 1.0*^#^ |
| hyodeoxycholic acid | 2.4 ± 1.1 | 6.0 ± 2.2* | 4.0 ± 1.7 |
| β-muricholic acid | 66.1 ± 28.9 | 98.2 ± 36.2 | 64.8 ± 17.1 |
| ω-muricholic acid | 21.8 ± 8.1 | 27.6 ± 6.4 | 26.9 ± 5.8 |
|  |  |  |  |
| **total fecal bile salts** | **10.9 ± 1.5** | **9.1 ± 0.8** | **10.1 ± 1.1** |
| α-muricholic acid | 0.4 ± 0.1 | 1.0 ± 0.2* | 0.6 ± 0.1*^#^ |
| deoxycholic acid | 4.8 ± 1.6 | 2.5 ± 0.7* | 4.2 ± 1.3^#^ |
| cholic acid | 2.9 ± 1.6 | 1.1 ± 0.7 | 2.0 ± 1.6 |
| hyodeoxycholic acid | 0.2 ± 0.2 | 0.4 ± 0.2 | 0.4 ± 0.2 |
| β-muricholic acid | 0.5 ± 0.1 | 1.0 ± 0.2* | 0.5 ± 0.2^#^ |
| ω-muricholic acid | 2.2 ± 0.4 | 3.0 ± 0.8 | 2.4 ± 0.3 |
|  |  |  |  |
