# Supplementary material for: Hepatic Farnesoid X-Receptor Isoforms α2 and α4 Differentially Modulate Bile Salt and Lipoprotein Metabolism in Mice
Source: PLoS One. 2014 Dec 15;9(12):e115028. doi: 10.1371/journal.pone.0115028 (PMC4266635; doi:10.1371/journal.pone.0115028)
Supplement: S3 Table — Differentially expressed genes of explorative Illumina microarray of livers of 0.5% cholic acid-fed stably transduced scAAV- FXRα2- and scAAV-FXRα4-FXR knock-out mice. Livers of 0.5% cholic acid-fed stably transduced scAAV- FXRα2- and scAAV-FXRα4-FXR knock-out mice were used for explorative microarray analysis. Listed top-15 genes and interesting targets, which showed statistically significant differences with a False Discovery Rate (FDR) <10%. “Fold change” represents the fold difference in expression between livers of stably transduced scAAV-FXRα2-and scAAV-FXRα4 FXR knock-out mice (both n = 6). The genes represented in bold are involved in bile salts and lipid metabolism. (DOCX) [file pone.0115028.s004.docx]

**Table S3. Differentially expressed genes of explorative Illumina microarray of livers of 0.5% cholic acid-fed stably transduced scAAV- FXRα2- and scAAV-FXRα4-FXR knock-out mice**

Livers of 0.5% cholic acid-fed stably transduced scAAV- FXRα2- and scAAV-FXRα4-FXR knock-out mice were used for explorative microarray analysis. Listed top-15 genes and interesting targets, which showed statistically significant differences with a False Discovery Rate (FDR) < 10%. “Fold change“ represents the fold difference in expression between livers of stably transduced scAAV-FXRα2-and scAAV-FXRα4 FXR knock-out mice (both n=6). The genes represented in bold are involved in bile salts and lipid metabolism.

| **Upregulated in FXRα2** | | | **Upregulated in FXRα4** | | |
| --- | --- | --- | --- | --- | --- |
| **rank** | **gene** | **fold change** | **rank** | **gene** | **fold-change** |
| 1 | *Ptgds* | 8.77 | 1 | *Cyp2a4* | 4.03 |
| 2 | *Lcn13* | 7.39 | 2 | *Cyp2b9* | 2.72 |
| 3 | *Cyp4a12b* | 4.29 | 3 | ***Cyp17a1*** | 2.57 |
| 4 | *Cyp2d9* | 3.04 | 4 | *Sult2a2* | 2.36 |
| 5 | *Tmem141* | 3.00/2.83 | 5 | *Ccbl1* | 1.84/1.83 |
| 6 | *Cyp4a12a* | 2.80 | 6 | *Cyp2b10* | 1.82 |
| 7 | *G6pc* | 2.43 | 7 | *Cyp2b23* | 1.74 |
| 8 | *Esam* | 2.08 | 8 | *Nnmt* | 1.74/1.66 |
| 9 | *Mup2* | 2.07 | 9 | *D14Ertd449e* | 1.73 |
| 10 | *Dct* | 2.05 | 10 | *Sds* | 1.62 |
| 11 | *Mup21* | 2.03 | 11 | *Prepl* | 1.62 |
| 12 | *Adssl1* | 1.99 | 12 | *Hhex* | 1.61 |
| 13 | *Mup20* | 1.96 | 13 | *Gstt3* | 1.60 |
| 14 | *Mug2* | 1.95/1.87 | 14 | *Serpina6* | 1.53 |
| 15 | *Cyp4a10* | 1.81 | 15 | *Pah* | 1.52 |
| 27 | ***Ostb*** | 1.74 | 32 | ***Abcg8*** | 1.39 |
| 36 | ***Cyp7b1*** | 1.68 | 46 | ***Vldlr*** | 1.34 |
| 90 | ***Abcb11*** | 1.37 | 117 | ***Cyp7a1*** | 1.23 |
| 213 | ***Apoc2*** | 1.22 | 134 | ***Cyp8b1*** | 1.21 |
